# Mitochondrial Complex I Core Protein Regulates cAMP Signaling via Phosphodiesterase Pde2 and NAD Homeostasis in *Candida albicans*

**DOI:** 10.3389/fmicb.2020.559975

**Published:** 2020-11-26

**Authors:** Xiaodong She, Lulu Zhang, Jingwen Peng, Jingyun Zhang, Hongbin Li, Pengyi Zhang, Richard Calderone, Weida Liu, Dongmei Li

**Affiliations:** ^1^Institute of Dermatology, Chinese Academy of Medical Sciences (CAMS) & Peking Union Medical College (PUMC), Nanjing, China; ^2^Department of Microbiology & Immunology, Georgetown University Medical Center, Washington, DC, United States; ^3^Department of Dermatology, Jiangsu Province Hospital of Traditional Chinese Medicine, Nanjing, China; ^4^Department of Dermatology, The First Affiliated Hospital of Kunming Medical University, Kunming, China; ^5^Sport Science Research Center, Shandong Sport University, Jinan, China; ^6^Center for Global Health, School of Public Health, Nanjing Medical University, Nanjing, China

**Keywords:** mitochondrial complex I, ergosterol synthesis, NADH/NAD^+^ redox state, PDE2 regulation, *Candida albicans*

## Abstract

The cyclic adenosine 3′,5′-monophosphate (cAMP)/protein kinase A (PKA) pathway of *Candida albicans* responds to nutrient availability to coordinate a series of cellular processes for its replication and survival. The elevation of cAMP for PKA signaling must be both transitory and tightly regulated. Otherwise, any abnormal cAMP/PKA pathway would disrupt metabolic potential and ergosterol synthesis and promote a stress response. One possible mechanism for controlling cAMP levels is direct induction of the phosphodiesterase *PDE2* gene by cAMP itself. Our earlier studies have shown that most single-gene-deletion mutants of the mitochondrial electron transport chain (ETC) complex I (CI) are hypersensitive to fluconazole. To understand the fluconazole hypersensitivity observed in these mutants, we focused upon the cAMP/PKA-mediated ergosterol synthesis in CI mutants. Two groups of the ETC mutants were used in this study. Group I includes CI mutants. Group II is composed of CIII and CIV mutants; group II mutants are known to have greater respiratory loss. All mutants are not identical in cAMP/PKA-mediated ergosterol response. We found that ergosterol levels are decreased by 47.3% in the *ndh51*Δ (CI core subunit mutant) and by 23.5% in *goa1*Δ (CI regulator mutant). Both mutants exhibited a greater reduction of cAMP and excessive trehalose production compared with other mutants. Despite the normal cAMP level, ergosterol content decreased by 33.0% in the CIII mutant *qce1*Δ as well, thereby displaying a cAMP/PKA-independent ergosterol response. While the two CI mutants have some unique cAMP/PKA-mediated ergosterol responses, we found that the degree of cAMP reduction correlates linearly with a decrease in total nicotinamide adenine dinucleotide (NAD) levels in all mutants, particularly in the seven CI mutants. A mechanism study demonstrates that overactive *PDE2* and cPDE activity must be the cause of the suppressive cAMP-mediated ergosterol response in the *ndh51*Δ and *goa1*Δ. While the purpose of this study is to understand the impact of ETC proteins on pathogenesis-associated cellular events, our results reveal the importance of Ndh51p in the regulation of the cAMP/PKA pathway through Pde2p inhibition in normal physiological environments. As a direct link between Ndh51p and Pde2p remains elusive, we suggest that Ndh51p participates in NAD homeostasis that might regulate Pde2p activity for the optimal cAMP pathway state.

## Introduction

Candidiasis is the fourth most common cause of bloodstream infections (BSI) in United States hospitals (Berman and Sudbery, 2002). Other types of candidiasis are mucosal oral or vaginal candidiasis, whose predisposing factors differ from those associated with BSI. For several reasons, including azole resistance and poor diagnosis assays, candidemia remains a disease with high mortality. A strategy for the development of novel antifungals has been to identify new antifungals that act against fungal- or even *Candida*-specific targets (Gintjee et al., 2020; Li et al., 2020). The current antifungal pipeline on specific metabolic targets includes olorofim (nucleic acid synthesis) (Oliver et al., 2016), fosmanogepix (MGX) [glycosylphosphatidylinositol (GPI) synthesis] (Alkhazraji et al., 2019), and arylamidine T2307 (mitochondria) (Yamashita et al., 2019).

Our recent efforts have focused on the identification of mitochondrial targets that have specificity for fungi – almost exclusively subunit proteins of electron transport chain (ETC) complex I (CI). To this end, we have identified two CI subunit proteins, Nuo1p and Nuo2p, as well as a CI regulator, Goa1p, that are fungal specific or CTG specific. The CTG clade contains a few opportunistic pathogenic yeasts (mostly *Candida* spp. but not *Saccharomyces cerevisiae*) that encode the CUG codon as a serine instead of a leucine. Other CTG-specific subunits of ETC CIII and CIV have also been recently reported (Sun et al., 2019). Fungal specificity, once established, should lead to the identification of functions that relate to the contribution of these proteins to host immune responses and pathogenesis. We have constructed knockout strains in the genes encoding the proteins described above. Among functional assignments, ETC mutants have a number of defects related to oxidative metabolism [ATP synthesis, oxygen consumption, reactive oxygen species (ROS) sensitivity], aging, survival in phagocytes, and virulence in mice and *Drosophila melanogaster*. We identified functions of the fungal-specific CI subunits that include cell wall polysaccharide synthesis. A third subunit protein of interest to us is a CI protein that is broadly conserved among species: Ndh51p. The use of this knockout strain has allowed us to categorize conserved functions and compare them to those having fungal-specific functions.

Genomic comparisons of *Candida albicans* and *S. cerevisiae* reveal many similar signaling pathways that regulate cellular processes, including cell cycle, morphogenesis, stress adaptation, and energy metabolism. The equilibrium between energy-generating and energy-depleting biosynthetic events is modulated by several conserved signal pathways, including Snf1 (Ulery et al., 1994; Mayer et al., 2011) and cyclic adenosine 3′,5′-monophosphate (cAMP)-activated protein kinase A (PKA). The Snf1 kinase pathway of *C. albicans* is a homolog of the mammalian AMPK for energy regulation. We have demonstrated that Snf1 is phosphorylated either during mitochondrial ATP insufficiency or during the addition of cAMP (Zhang et al., 2018). In contrast to the stress-responding Snf1 kinase pathway, the cAMP/PKA pathway functions mainly to regulate nutrient metabolism by coordinating energy consumption with cell activities such as fungal germination, cell cycling, and ergosterol biosynthesis under physiological conditions. However, mitochondrial ETC features of *C. albicans* are different from those of *S. cerevisiae* since the latter yeast species entirely lacks a mitochondrial CI. Also, *S. cerevisiae* is a Crabtree-positive organism that undergoes fermentation in the presence of oxygen, while *C. albicans* is Crabtree negative and uses oxygen for energy production.

The essential role of the Ras-cAMP-PKA chain has been conserved evolutionarily since the progenitors of yeasts and mammals diverged (Kataoka et al., 1985). It is initiated by carbon-sensing proteins Ras1/2p and Gpr1/Gpa2p. Once the GTP-bound form of Ras1/2p is activated by the guanine nucleotide exchange factor (GEF) Cdc25p, adenylate cyclase (Cyr1p) converts ATP into cAMP that initiates PKA (Tpk1p/Tpk2p) activation. The cAMP/PKA pathway regulates mitochondrial CI but not CII respiration in isolated liver (Lark et al., 2015). However, the coordination of cAMP/PKA signaling with CI function is not well understood in *C. albicans*. A number of studies have suggested a link between the cAMP/PKA pathway and mitochondrial oxidative phosphorylation (OXPHOS) in *S. cerevisiae* (Dejean et al., 2002; Hlavatá et al., 2008), in which enzyme content, ROS, the antioxidant defense system, and mitochondrial protein import are all significantly disrupted by cAMP/PKA dysfunction (Chevtzoff et al., 2005; Feliciello et al., 2005; Schmidt et al., 2011). As *S. cerevisiae* lacks CI, one can imagine that cAMP/PKA regulation of energy production in *C. albicans* would be different from *S. cerevisiae*. Structurally, *C. albicans* contains two Ras homologs Ras1p and Ras2p with redundant functions, and Ras2p has been further identified as part of the feedback inhibition of PKA (Zhu et al., 2009; Dong and Bai, 2011). In contrast to the multiple effectors in mammalian cells, Ras1p and Ras2p appear to have a single effector (Cyr1p) in *C. albicans*. To avoid an overreaction in cAMP/PKA production, cAMP conversion to AMP is catalyzed by the enzyme phosphodiesterases (PDE) Pde1p and Pde2p in *C. albicans*. When mitochondrial activity is positively regulated by the RAS/PKA pathway (Dejean et al., 2002), the activity of this pathway is largely dependent on the cAMP levels in the cells (Chevtzoff et al., 2005). Intracellular cAMP levels are constantly maintained but still spike during the lag phase in both *S. cerevisiae* and *C. albicans* (van der Plaat, 1974; Nikawa et al., 1987) – perhaps as a precursor to the acceleration of carbon metabolism.

We have chosen to focus upon ergosterol synthesis because of its contributions to cell membrane stability and because ergosterol synthesis is the target of the triazole antifungals. We noted that 10 out of 12 ETC CI mutants, including *ndh51*Δ (Sun et al., 2013), are hypersensitive to fluconazole (FLC). Unlike those fungal-specific proteins mentioned above, conserved Ndh51p in all eukaryotes is a core protein of CI that exerts the critical function of NADH oxidation by binding to NADH and participating in electron transfer (Gabaldón et al., 2005). Downregulated ERG and efflux pump activity have been used to explain azole sensitivities in *goa1*Δ and *ndh51*Δ (Sun et al., 2013; She et al., 2016). While *ERGs* are also seen to be downregulated in two other CI mutants, *nuo1*Δ and *nuo2*Δ, but to a lesser extent, the expressions of *FLU1* and *MDR1* (the major facilitator gene superfamily) were unexpectedly increased. It should be noted that the CIII and CIV mutants are also sensitive to FLC (Sun et al., 2019). However, the expression of *ERG* genes and drug efflux pumps *CDR1* and *CDR2* (ABC family members) have been seen to be normal in CIII and CIV mutants (Sun et al., 2019). These results suggest that the mechanisms for FLC sensitivity in these respiration mutants are different.

**FIGURE 1:**
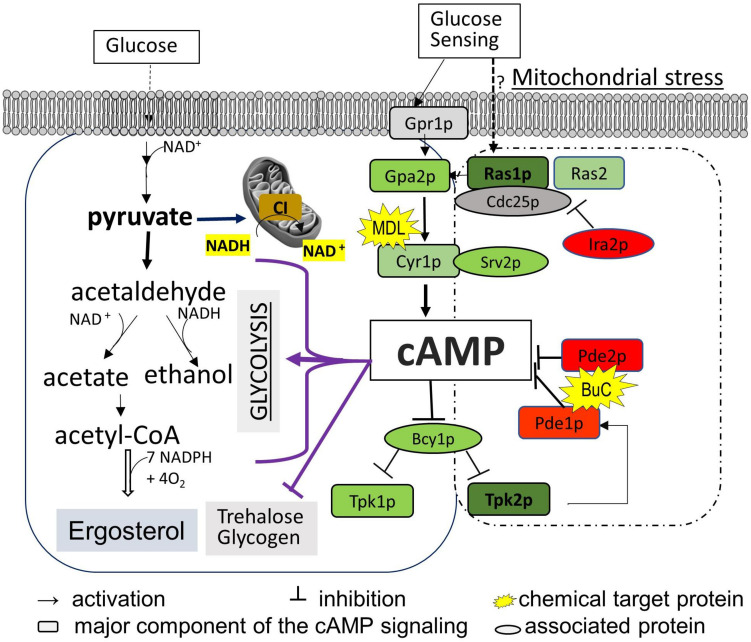
Diagram of the cAMP/PKA nutrient signaling pathway and two downstream effectors (ergosterol synthesis and trehalose accumulation) in *C. albicans*. Glucose metabolism in the mitochondria and cytosol depends on total NAD, which is constantly monitored by the cAMP/PKA pathway (left box). The major events in glycolysis that also require the NAD coenzyme are shown to highlight the possible affected steps for cAMP reduction observed in mitochondrial CI mutants such as *ndh51*Δ. We assume that Ras1-Tpk2 (in bold) activation harmonizes mitochondrial NAD metabolism and also actuates Pde1 to avoid an overactive cAMP/PKA response. The cAMP/PKA proteins are presented in green, and feedback control proteins are marked in red. MDL represents MDL-12330, which inhibits adenylyl cyclase for cAMP synthesis; BuC represents bucladesine, a cell membrane-soluble cAMP analog. Ergosterol content undergoes a change under either of BuC or MDL.

In order to determine the contribution of ergosterol synthesis to FLC sensitivity and to understand how cAMP/PKA-mediated ergosterol synthesis was affected by the mitochondrial ATP-generating process, we compared cAMP content and ergosterol abundance in two groups of ETC mutants with variable respiration defects and *ERG* profiles. In addition, trehalose and coenzyme nicotinamide adenine dinucleotide (NAD) content were used to estimate the degree of metabolic disorder due to abnormal cAMP pathway and NADH oxidation in the mitochondria. We employed two distinct testing models: the first is based on using gene-deleted strains of ETC [CI subunit mutants compared to that of wild type (WT) and other ETC complex mutants] during the early stationary phase of YP-glucose and YP-glycerol growth; the second model measures the effects caused by a cAMP antagonist and agonist versus untreated controls. Through the expression of genes in the cAMP pathway and PDE activities in the ETC mutants, we are able to demonstrate the molecular mechanism of a CI subunit protein that affects cAMP regulation. Our broad objective is to understand the impact of ETC proteins on cAMP/PKA-mediated ergosterol synthesis. Figure 1 describes key events in cAMP/PKA-mediated ergosterol synthesis. For each mutant used, we focused upon ergosterol levels, NAD content, cAMP levels, PDE activity, and trehalose synthesis, all of which are related to CI activity in *C. albicans* in Figure 1.

## Materials and Methods

### Culture Media

#### Media and Chemicals

YPD (2% glucose, 2% peptone, and 1% yeast extract) and YPG (2% glycerol, 2% peptone, and 1% yeast extract) were used. Compounds purchased from Sigma-Aldrich include bucladesine (BuC) and MDL-12330A [MDL, *cis-N*-(2-phenylcyclopentyl)-azacyclotridec-1-en-2-amine hydrochloride] and were dissolved in DMSO for a stock preparation. The working solutions were diluted with medium or phosphate-buffered saline (PBS) (pH 7.0).

#### Strains

SN250 was used as the parental strain (WT) for nine mitochondrial ETC subunit mutants tested in this study. ETC mutant strains include CI subunit mutants (*nuo1*Δ, *nuo2*Δ, *ndh51*Δ, *mt3290*Δ, *mt4758*Δ, and *mt7590*Δ), CI regulator *goa1*Δ, and one gene each for CIII and CIV (Noble and Johnson, 2005; Bambach et al., 2009; She et al., 2015). CI mutants and CI regulator *goa1*Δ are denoted as CI mutants throughout the text and experiments described below. The mutant strain *qce1*Δ and mutant strain *pet111*Δ are derived from the CIII subunit gene *QCE1* and CIV subunit *PET111*, respectively (Sun et al., 2019). Each of these mutants displayed a compromised respiration and was unable to assemble in the individual ETC complex (Li et al., 2011; She et al., 2015; Sun et al., 2019).

#### Growth Conditions

All cultures were streaked on a YPD agar plate from −80°C stocks. One colony was added to 5 ml of YPD broth to initiate overnight yeast cultures (200 rpm, 30°C). Cultures in YPD at 12 h were centrifuged at 4°C, and cell lysates were prepared from 200 ml medium for metabolite measurement. Cells were washed twice with cold PBS and stored at −80°C. For the YPG medium, as the ETC mutants are incapable of growing in non-fermentable glycerol, cells from a YPD culture at 10 h were collected, washed with PBS, and then incubated in YPG broth for an additional 2 h before being harvested. The cells were washed and stored at −80°C. For other experiments, cells grown in YPD for 10 h were supplemented with each compound at the concentration indicated in the results section. After a 2- or 4-h treatment according to each individual experiment, cells were washed with cold PBS twice and stored at −80°C until analysis (see below).

### Ergosterol Measurements

#### Sample Preparation

Cells were thawed at room temperature (RT), then sonicated for 10 min at 4°C, and were treated with 25 ml of 400 g/L KOH methanol (85–90°C for 2 h) (Abidi, 2001). After cooling, samples were shaken for 1 min in 6 g of sodium phosphate. The upper layer was adjusted to 25 ml with MeOH/acetone/*n*-hexane (2:2:1) (Nagy et al., 2006) and then subjected to filtration with a 0.45-μm Millipore filter. The filtrate was dried at RT under nitrogen, and the residue was dissolved in 50 μl MeOH for HPLC-mass spectrometry (MS) analysis.

Sterols in 10-μl aliquots were separated on a C18 (250 mm × 4.6 mm, 5 μm) chromatographic column (Shiseido, Japan) at 30°C. The solvent gradient used in the mobile phase is MeOH/H_2_O (97:3, v/v) at a flow rate of 1.0 ml/min. Detection of ergosterol was achieved at 280 nm (Kesselmeier et al., 1985) using HPLC (Agilent-1260, Agilent Technologies, Santa Clara, CA, United States). Samples were compared to commercial ergosterol (Sigma). The quantitation of total sterol content was calculated as follows: sterol content = C × N × V/M (C, concentration; V, volume; N, dilution factor; and M, dry weight of cell lysate).

### Determination of cAMP, NADH, and NAD^+^ in *C. albicans* Lysates by HPLC-MS/MS

#### Sample Preparation

Samples were lyophilized and then ground into powders. Approximately 100 mg of sample from each strain was dissolved in 1 ml of water. The suspension was vigorously vortexed for about 30 min at 4°C and then centrifuged at 13,200 rpm for 4 min. A 50-μl aliquot of supernatant of each strain was mixed with 150 μl of acetonitrile. A mixture of 50 μl was aspirated and subjected to HPLC-MS/MS analysis.

#### Instrument and Reagents

All the standard compounds and chemicals for HPLC-MS/MS including methanol and acetonitrile were purchased from Sigma. The HPLC-MS/MS system consisted of Shimadzu LC-20AD and API 3200MD TRAP. MS was performed in electrospray positive ionization mode with mass ranges of 124–1,000 and 8–1,000 Da. Experimental parameters for HPLC and tandem MS are as follows.

#### Liquid-Phase Conditions

The column temperature was maintained at 35°C. Each 10 μl of the samples was injected onto a MSLab 45 + AA-C18 column (4.6 × 150 mm, 5-μm particle diameter) at a flow rate of 0.75 ml/min. Metabolites were separated by a linear gradient of an aqueous phase solution A, water (ammonium acetate), and organic phase solution B, acetonitrile (ammonium acetate, ammonia, and water). The gradient was as follows: 1–2 min, 100% A; 3–4 min, 90% A; 5–6 min, 50% A; 7–8 min, 5% A; and 9–10 min, equilibration with 100% A. The fraction collected from the HPLC was coordinated as 0–1, 1.1–3.0, 3.01–5.0, 5.01–8.0, and 8.01–11.0 min. The total analysis time, including the equilibration, was 12 min for each analysis.

#### MS Conditions

Sciex API 3200, a fully integrated triple quadrupole mass spectrometer with ESI electrospray ion source was used and multiple-reaction monitoring (MRM) was operated for quantitative metabolite analysis. Samples were infused continuously at 5 μl/min. An atomizing gas (50 psi) and auxiliary gas (60 psi) were used as the nebulizing gas with collision gas (CAD) in the medium. A constant flow of curtain gas (20 psi) around the electrospray needle was supplied for reducing surface tension. The collision chamber injection voltages for exit potential (CXP) and entrance potential (EP) were −3.0 and −10 V, respectively. The electrospray needle voltage was set to −4.0 kV, and the heated capillary tube was kept at 350°C.

#### Quantitative Analysis of Metabolites

Metabolites of samples were identified by their retention time and mass spectral analyses (MS and MS/MS spectra); concentrations of metabolites were based on accurate mass, retention time, and MS/MS information in accordance with the published guidelines for metabolomics studies (Sumner et al., 2007; Wang et al., 2018).

### *RAS* and *PDE* Gene Expression in *C. albicans*

The expressions of *RAS1*, *RAS2*, *PDE1*, and *PDE2* were measured by RT-PCR. All strains of *C. albicans* (WT and complex mutants) were prepared in YPD mid-log phase growth at 30°C. RNAs were obtained from each strain following glass bead shaking and phenol extraction at 65°C. The quality and concentration of RNAs were measured with a nano-spectrophotometer, and approximately 0.8 μg of RNA was used to prepare cDNA and the real-time PCR procedure of QIAGEN (OneStep RT-PCR Kit). The transcription level of each gene was normalized to 18S rRNA gene level. Data are presented as means ± standard deviations (SD). The 2^−ΔΔ*CT*^ (where CT is the threshold cycle) method of analysis was used to determine the fold change in gene transcription (She et al., 2013).

### cAMP PDE (cPDE) Activity Assay

The cPDE activity assay kit from BioVision Inc. (Milpitas, CA, United States) is normally used to measure the PDE activity in mammalian cells. We modify the manufacturer’s protocol in order to apply the method to fungal cells with cell walls. The assay specifically targets PDEs that degrade cAMP molecules. When AMP produced by cPDE activity in the sample is metabolized by the enzyme mix provided in the kit, a newly formed intermediate compound will react with a fluorescent probe to generate a fluorescent signal at Ex/Em = 538/587. Measurement of fluorescence [relative fluorescence units (RFU)] was assessed in kinetic mode for 30 min at 37°C, and the results were plotted for calculation of cPDE activity.

The cells were harvested from 12 h of growth in YPD or YPG broth by centrifugation at 5,000 × *g* for 10 min. After washing twice with PBS, 2 × 10^6^ cells in each strain were suspended in 200 μl of cPDE assay buffer supplemented with 200 μl of glass beads. Cell lysate was achieved by rigorous shaking at 4°C for 3 min and subsequent centrifugation. An aliquot of 20 μl of cell lysate (optimal concentration of cPDE in preliminary experiment) was used to measure cPDE activity in a black 96-well plate with a flat bottom. For each experimental set, an appropriate standard curve for cPDE activity was generated by plotting the resulting RFU values against AMP concentrations from 20 to 100 nM, and positive and negative controls are included in each assay. The calculation of cPDE activity (the difference of ΔRFU_S_ between two time points) of each sample was based on the observed slope (the linear portion of the plotted RFU post 12 min of substrate addition). The testing well without substrate (background control) for each sample was set up in parallel. After subtraction of the background control (ΔRFU_bk_) for the same time interval, the RFU changes between two time points (ΔRFU_S_ value) were applied in standard curve to determine the AMP generation (con_AMP_). The cPDE activity (pmol/min/ml, or μU/ml) was then calculated according to the following formula: cPDE activity = (con_AMP_/Δ*T* × *V*) × D, in which Δ*T* represents the time interval chosen for the ΔRFU_S_ value (in minutes), *V* is the sample volume (ml), and D is the dilution factor. The cPDE activity in each strain was also normalized by protein concentration in each testing sample and was presented as μU/ml/mg protein. Protein concentration was determined by the Bradford reagent assay.

### Statistical Analysis

All the data were analyzed by SPSS Statistics 17.0. For each assay, three replicates were analyzed with one-way analysis of variance (ANOVA) along with Dunnett’s test to calculate the statistical difference between means. The comparisons were performed against WT *C. albicans* in the YPD condition or each strain without treatment if not indicated otherwise. Significance was established at *p* = 0.05.

## Results

### Variations in Ergosterol Reduction in Respiratory Mutants

Fluconazole is an inhibitor of ergosterol synthesis. Inhibition of CI and CIII–CV in *C. albicans* increases the susceptibility to FLC in strains found in clinical settings, lab isolates, and even in strains with an FLC resistance phenotype (Sun et al., 2013). The ETC deletion mutants chosen in this study fall in two classes based on their O_2_ consumption rates (OCRs) (Sun et al., 2019). All CI mutants are categorized as Class I, which sustains a 30–35% level of WT OCR that is no longer sensitive to rotenone (CI inhibitor) but is still sensitive to the CIV inhibitor KCN. This means that the residual OCR in Class 1 mutants is not a product of CI respiration. The CIII mutant (*qce1*Δ) and CIV mutant (*pet111*Δ) belong to Class II – characterized by maintaining only ∼7% of WT OCR levels, where this residual OCR is no longer sensitive to KCN. Both classes of respiratory mutants are hypersensitive to FLC (Sun et al., 2013). However, there seem to be mutant-specific mechanisms for the FLC susceptibility because of their variable efflux pump activities (Sun et al., 2013, 2019; She et al., 2015). In contrast to other CI mutants, the *ndh51*Δ mutant exhibits more pronounced changes in *ERGs* for ergosterol synthesis (Sun et al., 2013).

**FIGURE 2:**
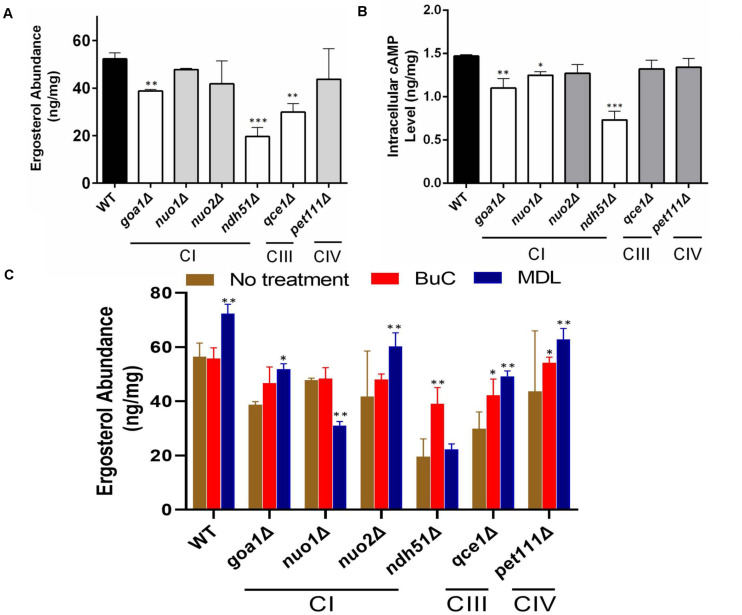
Reduction of ergosterol and cAMP levels varies with each respiration mutant, denoted by their affected ETC complexes: CI, CIII, or CIV null mutants. Ergosterol measurements from cell lysates extracted after 12-h growth in YPD at 30°C of HPLC **(A)** and cAMP levels from HPLC-MS/MS **(B)**. The ergosterol and cAMP content are presented in units of ng/mg cell lysate for each of the CI, CIII, and CIV mutants and compared with WT strain SN250. First, ergosterol reduction is more pronounced (than in WT) in the CI mutant *ndh51*Δ (*p* < 0.001), the CI regulator mutant *goa1*Δ (*p* < 0.01), and the CIII mutant *qce1*Δ (*p* < 0.01). Second, cellular cAMP concentration is markedly reduced in *ndh51*Δ (*p* < 0.001) but less reduced in *goa1*Δ (*p* < 0.01) and CI mutant *nuo1*Δ (*p* < 0.05). Third, the CIII mutant *qce1*Δ shows a 33% lower ergosterol level (compared to WT), but its cAMP reduction is less than 10%, while the CIV mutant *pet111*Δ shows a normal ergosterol level, with less than 10% cAMP reduction. **(C)** Ergosterol measurement under an agonist and an antagonist of cAMP suggests a negative feedback regulation of ergosterol synthesis upon inhibition of the cAMP/PKA pathway. Under a 4-h treatment with 50 μM BuC, the ergosterol reductions seen in the CI regulator mutant *goa1*Δ are restored (*p* > 0.05 versus WT). While no significant change occurs in WT, ergosterol increases 1.89- and 1.3-fold in the CI mutant *ndh51*Δ and CIII mutant *qce1*Δ, respectively, compared with their untreated baselines. However, the adenylyl cyclase inhibitor MDL at 50 μM concentration slightly elevates ergosterol content in WT and in most mutants (except for *ndh51*Δ and *nuo1*Δ). On the other hand, MDL suppresses ergosterol levels in *nuo1*Δ (*p* < 0.01) versus WT but that treatment cannot further suppress ergosterol levels in *ndh51*Δ. All experiments run in triplicate, with error bars generated from the triplicate data. *p* values are shown as “***” for <0.001, “**” for <0.01, and “*” for <0.05.

To determine the correlation between cell energy and azole susceptibility or ergosterol synthesis, we compared the ergosterol content in Class I mutants (three CI mutants: *nuo1*Δ, *nuo2*Δ, and *ndh51*Δ), the CI regulator mutant *goa1*Δ, and two Class II mutants (*qce1*Δ and *pet111*Δ). The ergosterol content was measured by HPLC in cellular lysates collected from early stationary phase growth. When compared to those in WT, significant reductions in ergosterol of 47.3, 32.8, and 23.7% were observed in *ndh51*Δ, *qce1*Δ, and *goa1*Δ (*p* values of <0.001 and <0.01), respectively, as shown in Figure 2A. Like GOA1, QCE1 is also a CTG lineage-specific protein. By contrast, ergosterol abundance remained relatively undisturbed upon deletion of the other two CI mutants (*nuo1*Δ and *nuo2*Δ) and *pet111*Δ. These results exhibit a lack of correlation between ergosterol synthesis and respiration activities. Apparently, mutant-specific ergosterol changes with one or more MFS (major facilitator superfamily) or CDR efflux pumps give rise to the common FLC sensitivity in these respiratory mutants. Furthermore, the greatest loss of ergosterol in *ndh51*Δ validates the greatest downregulation of the *ERG* gene family.

### Intracellular cAMP Levels Decrease in ETC Mutants, Especially in the CI Core Subunit Mutant *ndh51*Δ

The impact of cAMP levels on azole sensitivity (Jain et al., 2003) and ergosterol biosynthesis is well documented (Sardari et al., 2003). To confirm whether the reduction of ergosterol content in *ndh51*Δ, *qce1*Δ, or *goa1*Δ is correlated with cAMP levels in these mutants, cAMP levels in each of the six ETC mutants having variable ergosterol levels were assessed during early stationary phase growth in YPD (2% glucose). As shown in Figure 2B, the intracellular cAMP content of WT in overnight YPD culture was 1.47 ng/mg cell lysate. With the same carbon source and growth conditions, the cAMP level in *ndh51*Δ was 0.74 ng/mg cell lysate – 48% of the WT cAMP level (*p* < 0.001). In the other three CI mutants, an approximate 25% reduction was shown in *goa1*Δ (*p* < 0.01), and reductions of approximately 15% and 12% were found in *nuo1*Δ (*p* < 0.05) and *nuo2*Δ, respectively. At the same time, cAMP loss was less than 10% in the CIII mutant *qce1*Δ or the CIV mutant *pet111*Δ when compared to WT. The significantly reduced cAMP levels and losses in ergosterol in *ndh51*Δ and *goa1*Δ (Figure 2A) perhaps suggest a defect of cAMP-mediated ergosterol occurrence in these two CI mutants. However, this is not the case for ergosterol loss in *qce1*Δ.

### cAMP Agonist BuC Partially Restores Ergosterol Content in CI Mutants

To explain whether reduced cAMP is the cause of ergosterol reduction in *ndh51*Δ and *goa1*Δ shown above, we measured the ergosterol contents in each respiratory mutant under treatments of BuC and adenylyl cyclase inhibitor MDL-12330A (MDL) that have opposite effects on cAMP levels. BuC is a cAMP agonist that targets a PDE to increase cAMP in mammalian cells as shown in Figure 1. At 4 h post treatment with 50 μM BuC, cAMP slightly increased by 12–15% in all the mutants, which resulted in a significant restoration of ergosterol in *goa1*Δ (*p* > 0.05) and partial restoration of ergosterol levels in *ndh51*Δ and *qce1*Δ through 1.89- and 1.3-fold increases, respectively (Figure 2C). Under the same treatment, BuC unexpectedly decreased endogenous cAMP by 15% in WT, which leads to no change in its ergosterol level. Further studies showed that this cAMP reduction in WT remained over a range of BuC concentrations from 10 to 200 μM and does not operate in a dose-dependent manner (data not shown). These results suggest that a compensatory response, i.e., through an inhibition of cAMP generation or activation of cAMP degradation, may suppress the cAMP spike when PDE activity is inhibited by BuC. However, CI function-related PDE activity plays an important role in maintaining cAMP/PKA-mediated ergosterol or other downstream effectors.

When tested with cyclase inhibitor, MDL effectively decreased cAMP levels in mutants and WT, showing a dose-dependent manner over a range of 50 to 200 μM in WT (data not shown). We used 50 μM MDL to suppress cAMP in respiratory mutants. Contrary to our expectations, ergosterol levels were increased in WT strain under 50 μM MDL treatment, which was also evident in the two CI mutants *goa1*Δ and *nuo2*Δ and the CIII and CIV mutants (Figure 2C). On the other hand, ergosterol content was not further reduced in *ndh51*Δ under MDL treatment (*p* value > 0.05) but significantly decreased in MDL-treated *nuo1*Δ (*p* value < 0.01) when compared with each untreated baseline (Figure 2C). These results indicate that other pathways acted to oppose the downregulation of the cAMP-mediated ergosterol pathway in WT and most respiratory mutants. The insensitivity of *ndh51*Δ and apparent ergosterol reduction in *nuo1*Δ under MDL treatment suggest their possible roles in such compensatory responses for ergosterol biosynthesis under cAMP inhibition.

### Suppression of cAMP in *ndh51*Δ Increases Trehalose in *C. albicans*

**FIGURE 3:**
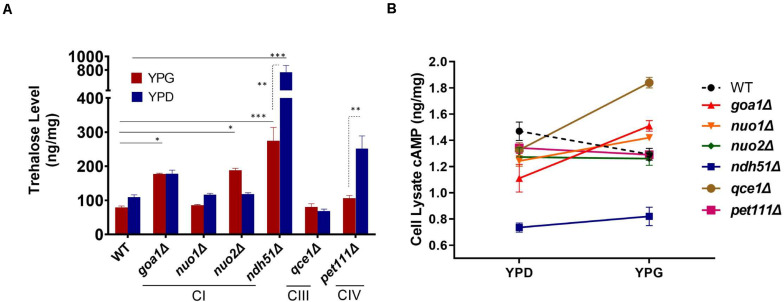
Metabolite disorders associated with the severity of cAMP reduction under mitochondrial stress. Growth conditions for YPG in ETC mutants initiated with 10-h YPD (2% glucose) culture due to their inability to utilize non-fermentable carbon sources. After washing twice with PBS, the cells from mutants and WT were transferred to YP-2% glycerol medium for 2 h before cell lysate preparation. **(A)** Trehalose content in *ndh51*Δ increases sevenfold (YPD) and threefold (YPG) over WT, which correlates with the greatest cAMP reduction in this mutant. Significant increases in trehalose are also shown in the CI regulator *goa1*Δ and *nuo2*Δ (under YPG) and in *pet111*Δ (under YPD). **(B)** Non-fermentable glycerol (YPG) growth represses cAMP levels and slightly decreases trehalose content in WT when compared with YPD. A contrary impact on the cAMP levels is seen in most ETC mutants during YPG growth. The changes in cAMP level between YPD and YPG in each mutant do not correlate with trehalose production in panel **(A)**. *p < 0.05, **p < 0.01, ***p < 0.001.

Trehalose is a stress protectant, along with glycogen, both acting to prepare yeast cells to enter the stationary growth phase under nutrient stresses. Trehalose also controls glycolytic flux and mitochondrial activity in yeast via the cAMP/PKA pathway to balance glycolysis with OXPHOS for ATP synthesis (Noubhani et al., 2009). In WT *Candida* cells, the trehalose level was seen to be slightly lower in YPG than in YPD (Figure 3A), which correlates with a 12% (1.29/1.47) cAMP reduction (Figure 3B). The lower trehalose levels under non-fermentable glycerol culture may help WT cells to increase mitochondrial activity during ATP synthesis, but this does not appear to be the case for mutants. For *ndh51*Δ grown in YPD, trehalose levels were sevenfold higher than for WT (*p* < 0.001) while cAMP was reduced by 48% in this mutant (Figures 3A,B). Withdrawal of glucose also appears to decelerate trehalose accumulation in the *ndh51*Δ strain; we observed a threefold higher trehalose level in YPG with only a slight cAMP elevation (Figure 3B). However, the increase in trehalose (2- to 2.5-fold) is marginally changed in other mutants such as CIV mutant *pet111*Δ under YPD and *goa1*Δ and *nuo2*Δ under YPG. Trehalose behavior under either medium for mutants is generally contrary to WT, betraying their metabolic disorders even in glucose-rich media. Nevertheless, there is no direct correlation between cAMP reduction and trehalose accumulation. The massive trehalose level in *ndh51*Δ is likely one of the consequent effectors of the suppressive cAMP/PKA pathway.

### NAD and cAMP Levels in Mitochondrial Mutants

**FIGURE 4:**
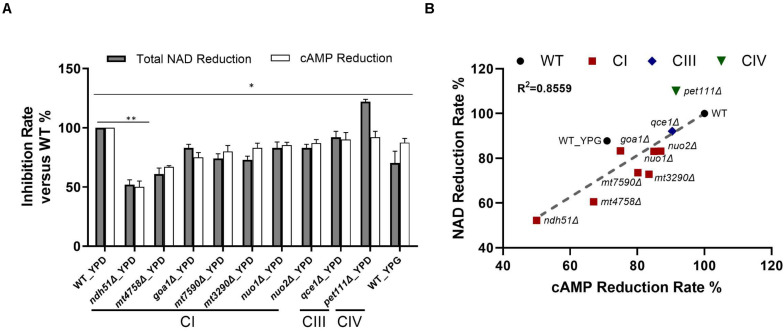
Direct correlation of total NAD with cAMP reduction levels. **(A)** The greatest NAD in the CI mutant *ndh51*Δ grown in YPD corresponds to a ∼50% cAMP reduction. The moderate NAD reductions shown in the CI mutant *mt4758*Δ and in the CI regulator mutant *goa1*Δ correspond to 32 and 25% cAMP reductions, respectively. A cAMP reduction of less than 10% in the CIII and CIV mutants reflects their respective abilities to maintain normal or even higher-than-normal NAD concentrations. **(B)** A strong direct (and linear) relationship between total NAD and cAMP reduction levels among CI, CIII, and CIV mutants (*R*^2^ = 0.8559). These results suggest a close relationship for ETC proteins with NAD metabolism and cAMP/PKA state during NADH oxidation in *C. albicans* mitochondria. Data are taken in triplicate, and comparisons are done between WT and each mutant in panels **(A,B)** under the same culture conditions. *p* values <0.001 are denoted as “**” for <0.01 and “*” for < 0.05.

The pyridine nucleotide cofactors NAD and NAD phosphate (NADP) are primary redox carriers in mitochondrial tricarboxylic acid (TCA) cycle, mitochondrial CI, and ergosterol biosynthesis. NAD represents the total pyridine nucleotide pool, while NAD^+^ and NADH are used to denote the specific respective oxidized and reduced forms. In the mitochondria, the electron donor NADH is oxidized by mitochondrial CI forming NAD^+^, which is then converted to NADH via the TCA cycle. To estimate whether dysfunctional CI affects the turnover of NADH, we measure NADH and NAD^+^ in all respiratory mutants described above. To further validate the correlation of NADH dehydrogenases of CI with cAMP and NAD, three additional CI mutants were included. The *mt7590*Δ and *mt4758*Δ are deletion mutants of ortholog core subunits NDUFS1 and NDUFS8 in humans (Lazarou et al., 2009), respectively. The *mt3290*Δ, which encodes the human ortholog NDUFS4, is not an enzymatic core subunit in any eukaryotic cell. The function of CI is carried by three domains: the electron transfer site, quinone-binding site, and proton translocation site. All three additional CI proteins, like Ndh51p, come from the electron transfer site. In addition, Ndh51p is one of the proteins to bind the NADH substrate (Gabaldón et al., 2005). We were not surprised to find that the operative structures of three mutants are similar, particularly to the role of Ndh51p. As shown in Figure 4A, reduction of cAMP levels in these additional CI mutants was greater than what we saw in *qce1*Δ and *pet111*Δ. We found that *mt4758*Δ and *mt7590*Δ showed 32 and 20% reductions, respectively, while the cAMP level in the non-core CI mutant *mt3290*Δ had a 17.5% reduction. In our previous study, *mt3290*Δ was one of the few CI subunit mutants that showed an MIC to FLC similar to that of WT cells (Sun et al., 2013).

For WT grown in 12 h YPD, a baseline level of 1.47 ng/mg cAMP correlated to 48.34 ng NAD per milligram of cell lysate with a 2.63 ratio of NAD^+^/NADH. In conjunction with a 12% reduction of cAMP in YPG, the total NAD molecules are reduced by 30% in WT, and the ratio of NAD^+^/NADH is significantly reduced to 0.96. Under the same 12-h YPD cultures, results for respiratory mutants showed that the relatively low levels of cAMP – especially in CI mutants – correspond numerically to a decreased total NAD (Figure 4B), of which *ndh51*Δ shows the greatest reduction in total NAD (48%) with the lowest cAMP level and an intermediate amount of total NAD reduction in other CI mutants corresponding to a respective intermediate reduction of cAMP level. The correlation coefficient is 0.8559 among all mutants as shown in Figure 4B.

The linear correlation between total NAD and cAMP content is less evident in each mutant when adjudged by changes of NAD^+^, NADH, or NAD^+^/NADH ratio as shown in Table 1. Although the cellular NAD^+^ level is one of the key factors regulating glycolytic speed, we found the greater reduction of NAD^+^ in all CI mutants and in the CIII mutant *qce1*Δ as well. As for NADH, we find that it is higher in those mutants with a more modest cAMP change, while the decreases only appear for the top three CI mutants (Table 1). The same suppression of NAD^+^ and NADH response in these top three CI mutants is also shown in WT under YPG conditions, suggesting that healthy electron transfer (in NADH metabolism) between CI and CIII releases a positive signal to activate the cAMP/PKA pathway to accelerate glycolysis and increase redox capability.

**TABLE 1:** Correlation of cAMP reduction with % changes in NAD^+^, NADH, and NAD^+^/NADH ratio in ETC mutants versus WT strain in YPD.

Strain (Complexes Mutant)	cAMP Change	NAD^+^ Change	NADH Change	NAD^+^/NADH ratio
*ndh51*Δ (CI)	−48.0%	−50.5%	−26.8%	1.04
*mt4758*Δ (CI)	−32.3%	−31.3%	−44.9%	1.41
*goa1*Δ (CI)	−25.1%	−13.1%	−24.2%	1.14
*nuo1*Δ (CI)	−15.0%	−36.9%	+ 34.5%	0.53
*nuo2*Δ (CI)	−12.3%	−24.3%	+ 9.4%	0.69
*mt3290*Δ (CI)	−17.5%	−35.4%	−7.5%	0.70
*mt7590*Δ (CI)	−19.8%	−36.2%	−2.9%	0.66
*qce1*Δ (CIII)	−9.5%	−38.8%	+ 71.4%	0.41
*pet111*Δ (CIV)	−8.2%	+ 29.8%	+0.4%	1.47
WT-YPG	−12.3%	−53.4%	+ 35.7%	0.43

### *ndh51*Δ Elicits an Unusually Strong Phosphatase PDE2 Response

The contribution of two RAS isomers and two isomers of PDE in the context of mitochondrial metabolic state is not known. The microarray data (Table 2) showed that CYR1 (adenylyl cyclase) expression was not affected in all mutants and that the overall downregulated cAMP/PKA pathway was more evident in CI mutants *ndh51*Δ and *goa1*Δ. Meanwhile, it also suggests that two RAS proteins and PDE proteins have opposite patterns in coordination with mitochondrial stress occurring in respiration mutants. Together with respiration defects observed in *ras1*Δ and *tpk2*Δ (data not shown), we proposed that Ras1p/Tpk2-activated Pde1 is a dominant axis relevant to the cAMP/PKA regulation for glycolysis in the cytosol and mitochondria under physiological conditions (Figure 1).

**TABLE 2:** Fold changes in gene expression in the cAMP pathway.

Genes	Systematicname	ETC mutant cAMP level (WT 1.47 ng/mg)					
		0.74	1.10	1.25	1.29	1.33	1.35
		
		*ndh51*Δ	*goa1*Δ	*nuo1*Δ	*nuo2*Δ	*qce1*Δ	*pet111*Δ
*RAS1*	*Orf*19.1760	−	−	–3.96	−3.93	−	
*CDC25*	*Orf*19.6926	–2.0	–2.0	−	−	−	−
*IRA2*	*Orf*19.5219	−	–2.78	2.96	2.4	−	
*RAS2*	*Orf*19.5902	–13.26	–2.78	33.93	45.30	−5.14	−5.68
*GPR1*	*Orf*19.1944	–2.05	–3.08	−	−	−	−
GPA2	*Orf*19.1621	−	−	–2.86	−2.28	−	−
*CYR1*	*Orf*19.5148	−	−	−	−	−	−
*SRV2*	*Orf*19.505	−	−	−	−2.31	−	−
*BCY1*	*Orf*19.2014	−	–2.07	–2.66	−2.32	−	−
*TPK1*	*Orf*19.4892	−	−	2.96	2.47	−	−
*TPK2*	*Orf*19.2277	−	–2.50	–3.47	−2.01	−	−
*PDE1*	*Orf*19.4235	−	–3.67	−	−	−	−
*PDE2*	*Orf*19.2972	−	−	3.05	2.12	−	−2.21

*“–”: change less than twofold.*

**FIGURE 5:**
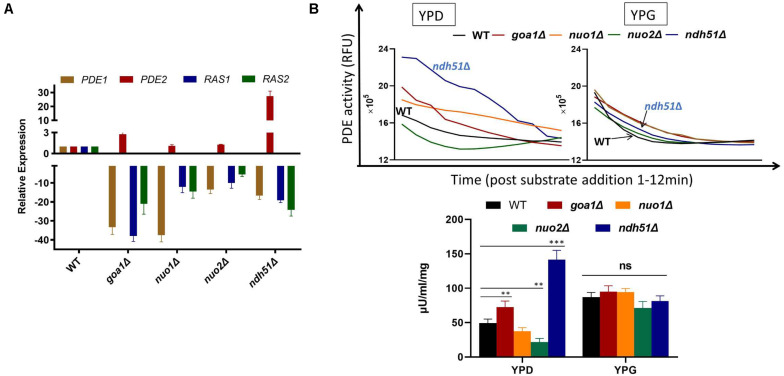
Validation of gene expression of two major G-proteins (*RAS1*/*RAS2*) and phosphodiesterases *PDE1*/*PDE2* by RT-PCR **(A)** and cPDE activity **(B)** in four CI mutants. **(A)** The four genes’ expression profiles illuminate the cAMP patterns in the four CI mutants. The relatively normal cAMP levels found in two of the CI mutants (*nuo1*Δ and *nuo2*Δ) correlate with a smaller scale of downregulation in *RAS1*, *RAS2*, and *PDE1* along with a normal *PDE2* expression. A severe cAMP reduction in *ndh51*Δ results from a >25-fold increase in *PDE2* expression while the other three genes are downregulated. A less than threefold increase in *PDE2* expression in *goa1*Δ, together with a significant level of downregulation for *PDE1*, results in a moderate cAMP reduction in this mutant. **(B)** Variation of cAMP-phosphodiesterase (cPDE) activity in four CI mutants in YPD; *ndh51*Δ shows the greatest activity. cPDE activity (slope of RFU change within 12 min after substrate addition) example shown in the top panel. An increased cPDE activity is shown for the mutants in YPD, but not in YPG. The bottom panel represents the cPDE activity from three experiments normalized to μU/ml/mg protein. A threefold higher cPDE activity is shown in *ndh51*Δ with *PDE2* upregulation and a 1.5-fold higher activity in *goa1*Δ versus WT. *p* values <0.001 are denoted as “***” and <0.01 as “**”.

In *S. cerevisiae*, Pde1p is involved in cAMP signaling induction, and Pde2p controls basal levels of cAMP, governing resistance of cells to various stresses, such as heat shock, nutritional starvation, and oxidative stress (Ma et al., 1999). To better explain cAMP reduction in CI mutants, the gene expressions of *RAS1*, *RAS2*, *PDE1*, and *PDE2* were analyzed in *ndh51*Δ and *goa1*Δ with the greatest (intermediate) reduction of cAMP using RT-PCR. The results were compared with those for *nuo1*Δ and *nuo2*Δ, which were predicated to have only minor effets on cAMP levels at transcription levels in the cAMP/PKA pathway. As seen in Figure 5A, the gene expressions of *RAS1*, *RAS2*, and even *PDE1* were significantly downregulated in the four CI mutants. However, *PDE2* was highly expressed (more than 25-fold) in *ndh51*Δ and only mildly enhanced (twofold) in *goa1*Δ, while it downregulated in *nuo1*Δ and *nuo2*Δ. Indeed, the net cAMP response in each of the four CI mutants matched their *RAS* and *PDE* expression patterns. For example, the highly upregulated *PDE2* repressed cAMP levels in *ndh51*Δ. The downregulated *PDE1* and *PDE2* and the reduced downregulated *RAS1* and *RAS2* help *nuo1*Δ and *nuo2*Δ to maintain a more compatible cAMP level. Together with the more modest *PDE2* upregulation, strongly downregulated *PDE1* and *RAS1* in *goa1*Δ compensate for and block further cAMP loss of the sort seen in *ndh51*Δ. The overactive *PDE2* mRNA in *ndh51*Δ is supported by a filamentous defect in *ndh51*Δ described previously (McDonough et al., 2002), as the *pde2*Δ mutant is hyperfilamentous and the constitutive overexpression of *PDE2* blocks bud-hypha transitions (Bahn et al., 2003).

### Elevation of PDE Activity in *ndh51*Δ

More than 10 PDEs with varying selectivity of cAMP and other cyclic nucleotides have been identified in mammals, where PDE4 is a predominant enzyme for cAMP degradation. Intracellular cAMP is synthesized from ATP by adenylyl cyclase and is inactivated by the hydrolytic cPDE superfamily enzyme. The cPDE activity assay kit used in this study is specific to cAMP-degrading enzymes. In *C. albicans*, two genes (*PDE1* and *PDE2*) are responsible for cAMP inactivation. At 12 h in YPD culture, we found that the total cPDE activity in *ndh51*Δ was threefold higher than in WT as shown in Figure 5B, which is correlated with the greatest upregulation of *PDE2* in Figure 5A. While the cPDE activity was also elevated in *goa1*Δ (1.5-fold), it was reduced in *nuo1*Δ and *nuo2*Δ by a factor of 1.3–2.3-fold. However, the divergent cPDE responses in these CI mutants become uniform upon glucose withdrawal. As seen in Figure 5B, the cPDE activity in each mutant turned out to be identical to that in WT in YPG. The total cPDE activities in these strains may be a result of the high-affinity Pde2p protein, which is supported by other observations. In particular, Pde1p of *C. albicans* hydrolyzes cGMP with a higher affinity than it did cAMP (Hoyer et al., 1994), suggesting a more important role for Pde1p in degrading cGMP than cAMP.

Taken together, the overexpression of *PDE2* and hyperactive cPDEs particularly in *ndh51*Δ and in *goa1*Δ to a lesser extent restricts the optimal level of cAMP during glucose metabolism. The highest ergosterol reduction and trehalose elevation seen in *ndh51*Δ reflect the downstream outcomes of suppressive cAMP/PKA. Therefore, the inhibition of PDE by BuC alleviates an originally low set of cAMP baselines, which leads to ergosterol restoration in *ndh51*Δ and *goa1*Δ. Given the metabolic disturbances in NAD levels seen in *ndh51*Δ and *goa1*Δ, we propose a CI protein Ndh51p-mediated Pde2p-cAMP regulation mode via an as-yet-undetermined NAD regulation mechanism. Furthermore, Goa1p likely utilizes the Ras1-PKA regulation model in order to coordinate TCA and lipid metabolism in *C. albicans*.

## Discussion

In eukaryotic cells, biochemical and genetic evidence strongly suggests a connection between mitochondrial status and activity of the cAMP pathway to regulate catabolism and anabolism. The interaction between the cAMP/PKA pathway and others harmonizes energy production with cellular activities according to nutrient availability and mitochondrial stresses. Through the use of a set of mitochondrial ETC complex mutants of *C. albicans*, the downstream responses of the cAMP pathway under disruption of NADH oxidation are investigated in this study. Through their different responses during cAMP-mediated ergosterol biosynthesis, cellular redox state, and glycolytic metabolites under different carbon sources, we uncover the roles of the ETC subunits on cAMP signaling in *C. albicans* that have not been well studied previously.

The Ras1-cAMP-PKA signaling pathway is critical for *C. albicans* animal model virulence (Rocha et al., 2001) in response to host ATP depletion and elevated CO_2_ levels (Tao et al., 2017). Loss of virulence is also one of the most common phenotypes of mitochondrial ETC CI mutants (Bambach et al., 2009; She et al., 2013, 2015; Huang et al., 2017). Using an array of respiratory mutants in this study, we observed a similar trend of cAMP reduction in CI mutants. All seven CI mutant (Class I) showed greater cAMP reduction than *qce1*Δ and *pet111*Δ (Class II) although Class II mutant has greater loss of respiration (Sun et al., 2019). Apparently, the ATP crisis is not a complete explanation for the severity of cAMP reduction.

The notion that Pde2p-mediated cAMP regulation is specific to Ndh51p arises from two observations in this study. First, *PDE2* expression increased >25-fold and cPDE activity increased three times in *ndh51*Δ. Second, the PDE inhibitor BuC partially restores cAMP ergosterol in mutants, including *ndh51*Δ. The PDE-regulated cAMP response is also supported by others’ observation that PDE inhibitors reverse the antifungal activity of azole drugs (Sardari et al., 2003). In *S. cerevisiae*, on the other hand, *pde1*Δ causes no change in cAMP levels, but *pde2*Δ reduces cAMP levels (Crauwels et al., 1997). In this study, the expression patterns of *RAS1*, *RAS2*, *PDE1*, and *PDE2* in four of the CI mutants showed that *PDE1* expression is in concordance with downregulation of *RAS1* and *RAS2* in all cases. It seems likely that Pde1p acts in parallel with Ras1p-PKA activation in terms of regulation of mitochondrial ATP synthesis. Since the *PDE2* response is opposite to that of *PDE1* in *ndh51*Δ or *goa1*Δ, two Pde1/Pde2 proteins in *C. albicans* may involve different regulation modes.

A high expression of *PDE2* in *ndh51*Δ explains the severe loss of cAMP in this mutant; moreover, a more downregulated *PDE1* moderates the degree of cAMP reduction due to slightly elevated *PDE2* and cPDE activities in *goa1*Δ. The severe or moderate cAMP reduction in *ndh51*Δ and *goa1*Δ explain the corresponding decrease in their ergosterol content. However, we are unable to apply the same reasoning to the 33% ergosterol reduction in *qce1*Δ. The smaller change in cAMP level in this CIII mutant thus suggests a cAMP-independent ergosterol biosynthesis mechanism where Qce1p might be involved. The elevation of ergosterol content in WT under MDL treatment (cAMP inhibitor) also supports the existence of this mechanism. As with ergosterol reduction, the trehalose accumulation is not corelated with cAMP content. A correlation coefficient greater than 0.85 between total NAD and cAMP in all nine tested mutants, on the other hand, justifies a linear correlation between NAD and cAMP. The correlation coefficient is even better when the CIV mutant is not included. These results suggest that total NAD is perhaps a bioenergetic marker to modulate the Ras1-cAMP-PKA regulation pathway in *C. albicans*. Indeed, the metabolism of NAD has emerged as a key regulator of cellular homeostasis (Nikiforov et al., 2015). Being a major component of both bioenergetic and signaling pathways, total NAD is ideally suited to regulate metabolism and major cellular events. In an early study, constant NAD levels in *C. albicans* were sustained during exponential growth and into the stationary growth phase (Chaffin et al., 1979). We assume that the unexplained persistence of high NAD after cessation of protein synthesis also implies that the NAD is upstream of cAMP, rather than being a downstream step in the cAMP pathway. In this case, when the depletion of glucose causes a slowdown in the turnover of NAD (metabolism) in the stationary phase, the accumulated NAD in turn would repress the cAMP response.

In this study, changes in NAD^+^ alone, or in NADH alone, or in the NAD^+^/NADH ratio do not correspond to any cAMP reduction in any mutant. This is somewhat noteworthy, since in mammalian cells, the NAD^+^/NADH ratio is an important marker to reflect the redox state of a cell for the metabolic activities during both catabolism and anabolism. Both the oxidized and reduced forms of NAD are maintained at significant concentrations, with the high NAD^+^/NADH ratio favoring the oxidative reaction through regulation of several key enzymes, including glyceraldehyde 3-phosphate dehydrogenase and pyruvate dehydrogenase. In conjunction with a range of 3–10 for the NAD^+^/NADH ratio in mammals, we observe a 2.63 ratio in the YPD growth in WT *C. albicans*, which then dropped to 0.9 in the non-fermentable glycerol medium due to the decrease in NAD^+^. The low NAD^+^/NADH ratio in the mutants indicates their insufficient glycolysis.

Our data exhibit a similar level of NAD^+^ decrease in all CI and CIII mutants, but not in the CIV mutant. The NAD^+^ reservoir is well known as a prerequisite condition for continued glycolysis in the cytosol. The significant reduction of NAD^+^ in the CI and CIII mutants represents a downregulated metabolic activity, perhaps including reduced ergosterol synthesis in CI mutants and the CIII mutant. In terms of NADH content, more than twofold decreases are seen in the top three CI mutants (*ndh51*Δ, *mt4758*Δ, and *goa1*Δ in Table 2), which had severe or intermediate cAMP reductions. The NADH reservoir is an important determinant not only for catabolic processes but also for some anabolic reactions, such as gluconeogenesis (Sistare and Haynes, 1985). Shortage of NADH suggests an additional role for the CI regulators Goa1p or NDUFS8 ortholog beyond their “major” CI enzymatic function. Perhaps, two proteins participate in NADH generation processes, i.e., the TCA cycle in the mitochondria, a process that utilizes non-fermentable carbon sources (such as amino acids and lipids) to generate ATP in the mitochondria. This hypothesis requires further investigation; however, the downregulated acryl-CoA carrier genes and the glycogenesis pathway in *goa1*Δ described in our previous study highlight possible roles of Goa1p in the regulation of NADH metabolism (Li et al., 2016).

Apparently, high levels of *PDE2* and cPDE activity are the causes of cAMP reduction in *ndh51*Δ; however, the invocation of a direct interaction (i.e., Ndh51p inhibiting Pde2p) seems somewhat contrived due to the different locations for the two proteins. The NAD-mediated model is suggested by the linear correlation evident between total NAD and cAMP. However, the current study cannot determine if the NAD loss in *ndh51*Δ is suffered in the cytosol or in the mitochondria. Whether NAD serves as a signal to link Ndh51p activity for NADH oxidization in the mitochondria with cytosol phosphodiesterase activity for controlling cAMP levels requires further investigation. To date, the knowledge of NAD synthesis or breakdown in *C. albicans* remains limited. Genomic BLAST suggests that this organism possesses a functional pathway for the endogenous synthesis of NAD from tryptophan and a salvage pathway from nicotinamide. BLAST analysis also identified an *NDT1* (mitochondrial NAD^+^ transporter of *S. cerevisiae*) ortholog – orf19.1393 – with an *E* value of 2.0e-91. Deletion of *NDT1* in *S. cerevisiae* decreases NAD^+^ and NADH content in mitochondria and reduces activity of mitochondrial NAD^+^-requiring enzymes (Todisco et al., 2006). Although the link between CI proteins and each NAD-maintaining mechanism above remains elusive, the report by Sporty et al. (2009) might offer an interesting area for future research, since they found that a functional salvage pathway is more important than the absolute NAD^+^ or NADH levels for life span extension under calorie restriction (CR) conditions. It should be noted that decreased life span is one common phenotype of CI mutants in our earlier studies (Chen et al., 2012; She et al., 2015).

## Conclusion

In conclusion, mitochondrial CI interacts with the cAMP/PKA signaling pathway that governs NADH metabolism in the mitochondria and redox potential in the cytosol, which promotes ergosterol synthesis and other virulence-related processes in *C. albicans*. A conserved mitochondrial CI subunit (Ndh51p) is required for maintenance of a high NAD potential and optimal concentration of cAMP via Pde2p inhibition. With regard to CI subunits, our data indicate a high degree of functional specificity. It is not surprising that the broadly conservative Ndh51p plays such an important role in cell membrane synthesis, when the fungal-specific subunits are so closely involved with cell wall assembly. This model also has implications in higher eukaryotes for cholesterol synthesis, since Ndh51p is a conserved protein in mammalian cells. The data we collect from ETC mutants also tempt us to explore an improved regimen for fungal infection by synergizing ergosterol inhibitors with cAMP antagonists.

## Data Availability Statement

The original contributions presented in the study are included in the article/supplementary material, further inquiries can be directed to the corresponding authors.

## Ethics Statement

No ethical clearance is required as no human or animal studies are presented in this study. All the experiments followed established biosecurity and institutional safety and ethical guidelines.

## Author Contributions

XS, DL, RC, and WL designed the experiments. XS, LZ, JP, JZ, PZ, and HL performed the experiment and collected the data. XS and DL designed and performed the analysis. DL, RC, and WL wrote the manuscript. All authors contributed to the article and approved the submitted version.

## Conflict of Interest

The authors declare that the research was conducted in the absence of any commercial or financial relationships that could be construed as a potential conflict of interest.
